# Normal serum protein electrophoresis and mutated IGHV genes detect very slowly evolving chronic lymphocytic leukemia patients

**DOI:** 10.1002/cam4.1510

**Published:** 2018-05-09

**Authors:** Jasmine Chauzeix, Marie‐Pierre Laforêt, Mélanie Deveza, Liam Crowther, Elodie Marcellaud, Paco Derouault, Anne‐Sophie Lia, François Boyer, Nicolas Bargues, Guillaume Olombel, Arnaud Jaccard, Jean Feuillard, Nathalie Gachard, David Rizzo

**Affiliations:** ^1^ Hématologie Biologique Centre Hospitalier Universitaire de Limoges Limoges France; ^2^ Faculté de Médecine de Limoges UMR CNRS 7276 CRIBL Limoges France; ^3^ Faculté de Médecine de Limoges EA6309 Limoges France; ^4^ Immunologie Centre Hospitalier Universitaire de Limoges Limoges France; ^5^ Hématologie Clinique et Thérapie Cellulaire Centre Hospitalier Universitaire de Limoges Limoges France

**Keywords:** Chronic lymphocytic leukemia, IGHV, prognosis, serum protein electrophoresis

## Abstract

More than 35 years after the Binet classification, there is still a need for simple prognostic markers in chronic lymphocytic leukemia (CLL). Here, we studied the treatment‐free survival (TFS) impact of normal serum protein electrophoresis (SPE) at diagnosis. One hundred twelve patients with CLL were analyzed. The main prognostic factors (Binet stage; lymphocytosis; IGHV mutation status; *TP53*,*SF3B1*,*NOTCH1,* and *BIRC3* mutations; and cytogenetic abnormalities) were studied. The frequencies of IGHV mutation status, cytogenetic abnormalities, and *TP53*,*SF3B1*,*NOTCH1,* and *BIRC3* mutations were not significantly different between normal and abnormal SPE. Normal SPE was associated with Binet stage A, nonprogressive disease for 6 months, lymphocytosis below 30 G/L, and the absence of the IGHV3‐21 gene rearrangement which is associated with poor prognosis. The TFS of patients with normal SPE was significantly longer than that of patients with abnormal SPE (log‐rank test: *P* = 0.0015, with 51% untreated patients at 5.6 years and a perfect plateau afterward vs. a median TFS at 2.64 years for abnormal SPE with no plateau). Multivariate analysis using two different Cox models and bootstrapping showed that normal SPE was an independent good prognostic marker for either Binet stage, lymphocytosis, or IGHV mutation status. TFS was further increased when both normal SPE and mutated IGHV were present (log‐rank test: *P* = 0.008, median not reached, plateau at 5.6 years and 66% untreated patients). A comparison with other prognostic markers suggested that normal SPE could reflect slowly advancing CLL disease. Altogether, our results show that a combination of normal SPE and mutated IGHV genes defines a subgroup of patients with CLL who evolve very slowly and who might never need treatment.

## Introduction

Chronic lymphocytic leukemia (CLL), the most frequent indolent B‐cell cancer with blood passage of tumor cells in the elderly in western countries, is characterized by a lymphocytosis exceeding five G/L comprising small circulating monomorphic round B lymphocytes, with constant infiltration of bone marrow and secondary lymphoid organs 1, 2. Diagnosis requires flow cytometry immunophenotyping of cells, usually based on the Matutes–Moreau score, which combines the expression of CD5 and CD23, weak surface membrane immunoglobulin levels and weak or absent FMC7, CD22, or CD79b expression. CLL is typically characterized by a Matutes score of 4 or 5 3, 4. Disease progression is heterogeneous with overall survival (OS) ranging from a few years to decades.

Because some patients with CLL may have a life expectancy as long as healthy subjects of the same age without treatment while others will rapidly evolve, it has been recognized for a long time that finding reliable prognostic factors is necessary. The first and most universal clinical and biological predictive tools are the Binet and Rai staging systems that predict the clinical course 5, 6. For example, patients with Binet Stage A CLL exhibit good treatment‐free survival (TFS), with a life expectancy close to that of healthy subjects of the same age. However, it is well known that the progression of these patients is rather heterogeneous 7.

Almost twenty years after the publication of Binet and Rai stages, Damle et al. 8. and Hamblin et al. 9. showed that a greater than two percent mutation rate of the variable rearranged region of the immunoglobulin heavy chain gene (IGHV) predicts good overall survival of patients with CLL. In the early 2000s, various cytogenetic prognostic markers were identified, including isolated del(13q), which predicts very good overall survival, and del(17p), which is reliably associated with resistance to fludarabine. High‐throughput sequencing of the CLL genetic landscape revealed several new gene mutations associated with a poor prognosis. For example, *NOTCH1* is associated with Richter syndrome, and the *SF3B1* mutation also seems to predict fludarabine resistance 10. Most of these genetic markers are in fact predictive of more rapid disease progression, treatment resistance, or transformation, and very few of them identify patients who will never need to be treated. Mutated IGHV and the deletion of the long arm of chromosome 13 (del(13q)) may be the only markers that predict prolonged TFS 11, 12, 13, 14.

To assess the cytogenetic and mutational status of CLL, each case requires high levels of technical and medical expertise, and the process is time consuming and costly 15. To circumvent these difficulties, various other biological parameters, such as serum soluble CD23, CD38, and ZAP70 expressions, have been reported as prognostic markers 16. However, as exemplified for antibodies detecting ZAP70, published reagents and/or methods for the detection and quantification of these markers are rather diverse 17. To date, these markers are rarely used in the routine staging and follow‐up of patients with CLL.

Universal biological analyses prescribed for the initial staging of patients with CLL include serum protein electrophoresis (SPE). SPE detects either hypogammaglobulinemia, which is supposed to predict infection risk 18 and is likely to be related to tumor burden 19, or immunoglobulin peaks. The prevalence rates of hypogammaglobulinemia and monoclonal paraprotein are 25% 20 and 11% 21, respectively. The clinical significance of abnormal serum protein electrophoresis has been previously studied, showing a worse prognosis in the presence of serum paraprotein 19, 22. The impact of hypogammaglobulinemia is controversial regarding either infection risk, TFS, or OS 20, 23, 24. As most publications only report on the significance of hypogammaglobulinemia or the monoclonal component alone, the prognostic value of a normal gamma globulin serum profile (normal SPE) per se has not been reported.

Here, we studied the correlation of normal SPE with TFS and its relationship with other variables (Binet Stage, biological parameters, and genetic characteristics). We found that normal SPE is an independent good prognostic variable and that the combination of normal SPE and mutated IGHV genes predicts patients with excellent TFS who are likely to have very slowly progressing CLL and who will never need treatment after diagnosis.

## Materials and Methods

### Patients

We analyzed 112 samples from patients with typical CLL, diagnosed between 2004 and 2016, and 93% were diagnosed between 2004 and 2012. By flow cytometry, their Matutes score was 4 or 5 in all cases. Inclusion criteria were based on the availability of biologic samples and concomitant serum protein electrophoresis with immunofixation electrophoresis when a peak was detected. Normal concentrations of gamma globulin ranged from 6 g/L to 11 g/L. The threshold for peak detection was 1 g/L of monoclonal immunoglobulin. Patients were classified according to the Binet Stage, nonprogressive/progressive disease (known stable disease for 6 months or not), the Rossi score, and their IGHV mutation status.

### DNA extraction

Genomic DNA was extracted from peripheral blood mononuclear cells using the QIAamp DNA Blood Mini Kit (Qiagen, Venlo, the Netherlands) according to the manufacturer's instructions.

### Sequencing of SF3B1, NOTCH1, TP53, and BIRC3

Patients were first screened for mutations in *SF3B1* exons 14, 15, 16, and 18 (NM_012433), *NOTCH1* exon 34 (NM_017617), and *BIRC3* exons 6 to 9 (NM_001165) by Sanger sequencing (BigDye Terminator cycle kit (Thermo Fischer Scientific, Waltham, MA) and ABI PRISM 3130xl Genetic Analyzer) after amplification of 50 ng genomic DNA. Sequences were analyzed with the Mutation Surveyor DNA Analysis Software V4.0 (SoftGenetics, State College, PA). Patients not screened by Sanger sequencing were assayed by high‐throughput sequencing (HTS) (Proton, Ion Torrent), using a panel that targeted the same regions. The *TP53* gene was analyzed by HTS in all cases. The panels were designed on the AmpliSeq designer platform (http://www.ampliseq.com). Libraries were constructed using the Ion AmpliSeq Library kit 2.0 (Thermo Fischer Scientific) according to the manufacturer's instructions. Variants were filtered to retain exonic mutations and to obtain a prediction score of pathogenesis by SIFT and/or CADD positive or unknown on both scores. Known mutations were identified by querying the COSMIC, 1000 genomes, and dbSNP databases.

### Immunoglobulin gene sequence analysis

Amplification of V, D, and J rearranged genes was performed using the Biomed‐2 strategy with FR1 and FR2 primers as previously described. Sequence analysis of VDJ segments was performed as previously described 25.

### Cytogenetics

Conventional cytogenetic and fluorescence in situ hybridization were performed according to conventional procedures. Quantitative multiplex PCR of short fluorescent fragment (QMPSF) was performed as described elsewhere 26.

### Flow cytometry

Multiparametric flow cytometry analysis was performed on heparinized blood samples as described elsewhere 27.

### Statistics

The chi‐square test was used to evaluate the difference between categorical covariates for the SPE subgroups. A lymphocytosis threshold of 30 G/L was used as determined previously 28. The effects of demographics (age and sex), Binet stage, lymphocytosis, genetics (IGHV mutation status, *SF3B1,* and *NOTCH1* mutations), cytogenetics (trisomy 12, del(11q), del(13q), and del(17p)), and serum parameter (SPE subgroups) covariates on treatment‐free survival (TFS) were examined using the Cox proportional hazard model 29. Briefly, each covariate was first tested in univariate analysis. All significant covariates with a *P*‐value below 0.20 after univariate analysis were included simultaneously in the multivariate model. The significance of variables in the final model was tested by a backward stepwise process using the likelihood ratio to evaluate the effect of omitting variables. The stability of the final model was validated by performing 1000 bootstrap samples.

## Results

To study the significance of normal serum protein electrophoresis in CLL, a series of 112 patients was selected. Their clinical and biological characteristics are shown in Table 1. Among them, 49 (44%) had a normal SPE in the year of diagnosis, and 63 (56%) had an abnormal SPE (hypogammaglobulinemia: 24%; gamma globulin peak: 32%). These two groups of patients with CLL were comparable in terms of age and sex, as well as hemoglobin concentrations and platelet counts.

**Table 1:** Patient characteristics at diagnosis (mean ± standard deviation is given for age, lymphocytosis, hemoglobin, and platelet counts)

	Total (*n* = 112)	Normal SPE (*n* = 49)	Abnormal SPE (*n* = 63)	*P*
Sex	Men: 63.5% (*n* = 66)	Men: 63.3% (*n* = 31)	Men: 58.7% (*n* = 37)	NS
	Women: 36.5% (*n* = 38)	Women: 36.7% (*n* = 18)	Women: 41,3% (*n* = 26)	
Age (years)	66.6 ± 10.5	65.7 ± 10.5	67.3 ± 10.5	NS
Binet				
Stage A	81.3% (*n* = 91)	95.9% (*n* = 47)	69.8% (*n* = 44)	χ^2^ test: *P* = 0.001
Stage B	15.2% (*n* = 17)	2.0% (*n* = 1)	25.4% (*n* = 16)	
Stage C	3.6% (*n* = 4)	2.0% (*n* = 1)	4.8% (*n* = 3)	
Nonprogressive disease for 6 months after diagnosis	79.5% (*n* = 89)	98.0% (*n* = 48)	65.1% (*n* = 41)	χ^2^ test: *P* < 0.0001
IGHV gene mutation (M‐CLL)	59.5% (*n* = 66)	61.2% (*n* = 30)	58.1% (*n* = 36)	NS
Lymphocytosis (G/L)	32.8 ± 63.6	18.2 ± 13.3	44.1 ± 82.5	NS
Lymphocytosis >30 G/L*	25.9% (*n* = 29)	14.3% (*n* = 7)	34.9% (*n* = 22)	*t* test: *P* = 0.017
Hemoglobin (g/dL)	13.7 ± 1.8	14.1 ± 1.5	13.5 ± 2.0	NS
Platelets (G/L)	212.2 ± 87.7	228.1 ± 89.1	199.7 ± 184.5	NS
Cytogenetic				
del(17p)	0.9% (*n* = 1)	2.1% (*n* = 1)	0.0% (*n* = 0)	NS
del(11q)	11.7% (*n* = 13)	8.3% (*n* = 4)	14.3% (*n* = 9)	
Trisomy 12	15.5% (*n* = 17)	10.4% (*n* = 5)	19.4% (*n* = 12)	
Isolated del(13q)	28.8% (*n* = 32)	32.7% (*n* = 16)	25.8% (*n* = 16)	
Mutations				
*NOTCH1*	8.9% (*n* = 10)	12.2% (*n* = 6)	6.3% (*n* = 4)	NS
*SF3B1*	11.6% (*n* = 13)	6.1% (*n* = 3)	15.9% (*n* = 10)	
*TP53*	7.1% (*n* = 8)	4.1% (*n* = 2)	9.5% (*n* = 6)	
*BIRC3*	2.7% (*n* = 3)	2.0% (*n* = 1)	3.2% (*n* = 2)	

Regarding the Binet stages of the patients, 39 of 41 patients (96%) with normal SPE were Binet stage A, while 19 of the 63 (30%) patients with abnormal SPE were Binet stage B or C (Table 1, *P* = 0.001, chi‐square test). Nonprogressive CLL (known stable disease for 6 months) was found in 98% of patients with normal SPE (48/49), while 65% (41/63) of the patients with abnormal SPE had stable disease (*P* = 2 × 10^−5^, chi‐square test). The number of patients with lymphocytosis above 30 G/L at diagnosis was significantly lower in patients with normal SPE (14.3% in normal SPE vs. 34.9% in abnormal SPE, Student's *t* test: *P* = 0.017) (Table 1).

With a threshold of a 2% mutation rate, the number of patients with mutated (M‐CLL) or unmutated (UM‐CLL) IGHV genes was comparable between normal and abnormal SPE (Table 1). Analysis of the IGHV gene repertoire was performed for IGHV gene rearrangements with a frequency over 5% among all patients with CLL of this series. Patients with a normal SPE exhibited significantly increased usage of IGHV1‐2. In contrast, the rearrangement of the IGHV3‐21 segment, associated with a poor prognosis, was overrepresented in patients with abnormal SPE and was completely absent in patients with normal SPE (Fig. S1).

The *BIRC3* and *NOTCH1* mutation rates were low and comparable between patients with normal and abnormal SPE (Table 1). Although not significant, the *SF3B1* mutation rate was lower in patients with normal SPE (6% vs. 16% for abnormal SPE, log‐rank test: *P* = 0.11). There were no differences for del(11q), trisomy 12, or isolated del(13q) (Table 1). The number of patients with del(17p) was too low to draw any conclusion.

**Figure 1:**
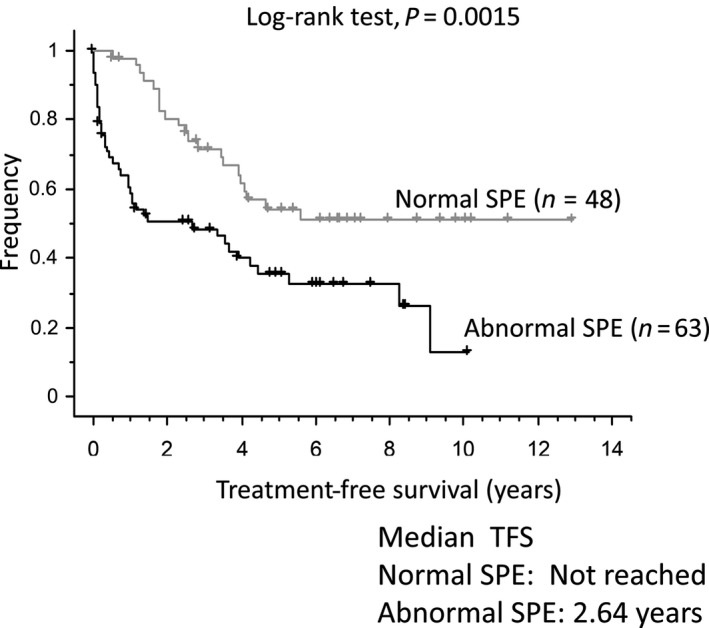
Kaplan–Meyer curves for treatment‐free survival according to SPE status.

We then studied TFS according to SPE. Patients with normal SPE had a significantly increased TFS, with a median that was not statistically reached. The plateau began at 5.6 years and was at 51% of cumulated TFS. Patients with abnormal SPE had a median TFS of 2.64 years with no plateau and had a comparable TFS to patients with either hypogammaglobulinemia or an immunoglobulin peak (Figs. 1 and S2). In comparison, the median TFS of patients with mutated IGHV was 8.2 years, and the plateau began at 8.4 years, with 48% of patients being treatment‐free (Fig. S3).

In addition to SPE status, the other significant variables after univariate analysis were hemoglobin levels, lymphocytosis, IGHV mutation status, *SF3B1* mutation, isolated del(13q), and del(11q). Criteria that integrated various variables, such as Binet stage or Rossi's score, were also highly significant (Table 2). Compared to normal SPE, the hazard ratio for treatment was 2.41 for abnormal SPE, a value that was compared to that of IGHV mutation status and of Rossi's score. For multivariate analysis, two Cox models were designed. The first model included the Binet stage, Rossi's score, IGHV mutation status, lymphocytosis, and SPE status, and the second model separated all biological criteria. Bootstrapping showed the stability of both models. Normal SPE was an independent variable in both. Other independent variables included the Binet stage, Rossi's score, and lymphocytosis for the first model, and IGHV mutation status and lymphocytosis for the second one (Table 3).

**Table 2:** Cox univariate analysis of TFS

	HR	LCI	UCI	*P*‐value
Binet stage				
Binet stage A (*n* = 91)	**1.00**			
Binet stage B (*n* = 17)	**21.44**	10.1	45.6	**<0.0001**
Binet stage C (*n* = 4)	**42.97**	11.2	164.6	**<0.0001**
Lymphocytosis				
<30 G/L (*n* = 83)	**1.00**			
≥30 G/L (*n* = 29)	**4.12**	2.44	6.96	**<0.0001**
IGHV mutation status				
M‐CLL (*n* = 66)	**1.00**			
UM‐CLL (*n* = 45)	**2.48**	1.49	4.13	**0.0005**
Hemoglobin				
>10 g/dL (*n* = 108)	**1.00**			
<10 g/dL (*n* = 3)	**14.37**	3.14	65.91	**0.0006**
SPE group				
Normal SPE (*n* = 49)	**1.00**			
Abnormal SPE (*n* = 63)	**2.41**	1.42	4.10	**0.001**
Rossi's score				
Very Low or Low (*n* = 69)	**1.00**			
Intermediate or high (*n* = 41)	**2.08**	1.25	3.45	**0.005**
SF3B1 status				
SF3B1 wild type (*n* = 99)	**1.00**			
SF3B1 mutation (*n* = 13)	**2.28**	1.18	4.42	**0.014**
Cytogenetics				
Normal FISH or QMPSF (*n* = 47)	**1.00**			
Isolated del(13)(q14) (*n* = 32)	**0.45**	0.24	0.83	**0.010**
Del(11q) (*n* = 13)	**2.15**	1.05	4.40	**0.036**
Trisomy 12 (*n* = 17)	**1.62**	0.86	3.07	**0.14**
Del(17p) (*n* = 1)^a^	**2.73**	0.37	19.96	**0.32**
Platelet count				
>100 G/L (*n* = 105)	**1.00**			
<100 G/L (*n* = 6)	**2.36**	0.85	6.56	**0.099**

HR, hazard ratio; LCI, lower confidence interval; UCI, upper confidence interval; *P*‐value, probability that the hazard ratio = 1 (null hypothesis).

a Excluded criteria because of low numbers.

**Table 3:** Statistically significant variables after Cox multivariate analysis of TFS (the two models were designed to include all nonoverlapping variables with a *P*‐value <0.20 after univariate analysis)

	1st Model								2nd Model							
	Multivariate analysis				Internal bootstrapping validation (mean)			% Bootstrap selection	Multivariate analysis				Internal bootstrapping validation (mean)			% Bootstrap selection
	HR	LCI	UCI	*P*‐value	HR	LCI	UCI		HR	LCI	UCI	*P*‐value	HR	LCI	UCI	
Binet stage																
Binet stage A (*n* = 91)	**1.00**				**1.00**			84.5								
Binet stage B (*n* = 17)	11.92	5.39	26.35	**<0.0001**	11.92	5.39	26.36									
Binet stage C (*n* = 4)	72.34	16.69	313.49	**<0.0001**	72.34	16.69	313.28									
Lymphocytosis																
<30 G/L (*n* = 83)	**1.00**				**1.00**			83.8	**1.00**				**1.00**			97
≥30 G/L (*n* = 19)	**2.53**	1.43	4.50	**0.002**	2.53	1.43	4.50		3.40	1.98	5.84	**<0.0001**	3.40	1.98	5.85	
SPE group																
Normal SPE (*n* = 49)	**1.00**				**1.00**			70	**1.00**				**1.00**			78
Abnormal SPE (*n* = 63)	**2.52**	1.43	4.43	**0.001**	**2.52**	1.43	4.43		**2.25**	1.30	3.88	**0.004**	**2.25**	1.30	3.88	
IGHV mutation status																
M‐CLL (*n* = 66)	**1.00**				**1.00**			67.5	**1.00**				**1.00**			85.5
UM‐CLL (*n* = 45)	**1.99**	1.15	3.44	**0.01**	**1.99**	1.15	3.45		**2.47**	1.47	4.17	**0.0007**	**2.47**	1.47	4.17	
Hemoglobin																
<10 g/dL (*n* = 108)									**1.00**							53
>10 g/dL (*n* = 3)									**17.10**	3.33	87.72	**0.0007**	**17.10**	3.34	87.68	

HR, hazard ratio; LCI, lower confidence interval; UCI, upper confidence interval; *P*‐value, probability that hazard ratio = 1 (null hypothesis).

**Figure 2:**
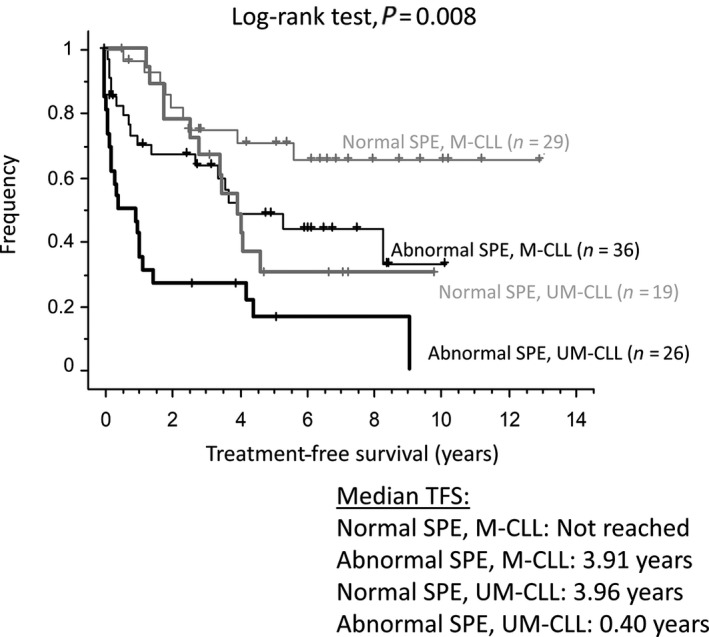
Kaplan–Meyer curves for treatment‐free survival according to SPE and IGHV mutation status.

We looked at the relationship between SPE and either the IGHV mutation status or Rossi's score. Patients with normal SPE and mutated IGHV had an excellent prognosis (median TFS not reached after 13 years of follow‐up, with a plateau at 5.6 years and 66% of accumulated TFS), while patients with normal SPE and unmutated IGHV genes or abnormal SPE and mutated IGHV genes had comparable and shorter TFS (median TFS at 3.96 years and 3.91 years, respectively, Fig. 2). The group with abnormal SPE and unmutated IGHV genes had a very short TFS (median TFS = 0.40 years). If restricted to the 84 patients with both stage A and stable CLL, the TFS curves of patients with normal and abnormal SPE were parallel for the first 2.6 years. However, a plateau was reached at 5.6 years only for patients with normal SPE and mutated IGHV genes (Fig. S4A). Indeed, among patients with mutated IGHV genes, the TFS curves of those with normal or abnormal SPE were clearly separated if the starting time point was translated from 0 to 2.6 years (Fig. S4B).

Normal SPE had no correlation with the TFS of patients with isolated del(13q) (very low‐risk Rossi's score, Fig. S5A) but was correlated with the significantly improved TFS of all other patients of Rossi's model (Fig. S5B and C). Moreover, even though their numbers were low, patients with normal SPE, mutated IGHV genes, and isolated del(13q) together had much longer TFS than the others (log‐rank test: *P* = 0.0295, Fig. S6).

Altogether, our results suggest that the combination of normal SPE with mutated IGHV genes identifies patients with a very slowly evolving CLL, and even though isolated del(13q) was not selected after multivariate analysis, its presence is likely to be an additional favorable parameter.

## Discussion

In this study, we showed that normal SPE is an independent good prognostic marker *per se* that predicts longer TFS in CLL, but most importantly, the combination of normal SPE and mutated IGHV genes defines a subgroup of patients with excellent TFS.

CLL has a highly variable natural course with overall survival ranging from a few years to several decades. As very well described by Binet and Rai 5, 6, the difficulty for the physician is to correctly predict disease evolution as it has a strong impact on patient medical care. It is remarkable that their scoring systems, based on a few simple clinical and biological parameters defined more than 30 years ago, are still valid and have not been supplanted by any of the “new” markers published after these princeps papers. Facing this plethora of markers, the current tendency is to search for simplicity 30, 31, 32. In that context, the Binet and Rai scoring systems are still the keystones of CLL staging at diagnosis. Additionally, only IGHV mutation status and del(17p)/*TP53* mutations are currently recommended to predict CLL progression and resistance to therapeutics 30, 31, 33.

Despite this apparent simplicity, within each group and mainly within good prognosis groups (Binet stage A or Rai stage 0–I), patient progression is rather heterogeneous. Most of the other markers proposed in CLL staging are associated with poor prognosis when present. In other words, the absence of the marker is not inevitably associated with the absence of disease progression. IGHV mutation status and chromosome 13 abnormalities are thus particular as it is the presence of somatic mutations in the variable regions of the immunoglobulin heavy chain genes or the presence of isolated del(13q) that predicts favorable evolution 13. Our results show that combining SPE results and IGHV mutation status improves the power of predicting a favorable disease course as the presence of normal SPE plus mutated IGHV genes is associated with very long TFS. Of note, even if the number of patients in this series was too low to form a firm conclusion, the presence of these two criteria combined with the presence of isolated del(13q) tended to define an even better prognosis group.

Hamblin et al. 9. and Opezzo et al. 34. have proposed that CLL with mutated IGHV genes could be a different disease with a more indolent course. Normal SPE appeared to be an independent parameter to predict longer TFS and was associated with Binet stage A. For mutated IGHV genes, the presence of normal SPE could also reflect a more indolent natural history. Supporting this hypothesis is the fact that various poor prognostic markers, such as the short‐term evolution of the disease, lymphocytosis over 30 G/L, or the IGHV3‐21 gene rearrangement, were almost always absent in patients with normal SPE. Based on their excellent TFS, patients with CLL harboring both normal SPE and mutated IGHV status might never require any specific treatment.

## Conflict of Interest

None declared.

## Supporting information


**Figure S1**. IGHV repertoire‐Only IGHV gene rearrangements with a frequency over five percent of the whole series are shown (Chi^2^ test: *P* = 0.021 for IGHV1‐2 and *P* = 0.0095 for IGHV3‐21).

**Figure S2**. Kaplan Meyer curves for treatment‐free survival for normal SPE, hypogammaglobulinemia and monoclonal peak.

**Figure S3**. Kaplan Meyer curves for treatment‐free survival according to IGHV mutation status.

**Figure S4**. Kaplan Meyer curves for treatment‐free survival (A) restricted to patients with non‐progressive CLL for 6 months and Binet stage A, according to SPE and IGHV mutation status. (B) Restricted to patients with non‐progressive CLL for 6 months, Binet stage A and mutated IGHV status, according to SPE, starting at 2.6 years).

**Figure S5.** Kaplan Meyer curves for treatment‐free survival according to SPE status and Rossi's score (A: very good Rossi's score. B: good Rossi's score. C: Intermediate Rossi's score).

**Figure S6**. Kaplan Meyer curves for treatment‐free survival for CLL patients with mutated IGHV genes, normal SPE and isolated del(13q) versus other patients of this whole series.

## References

[1] Swerdlow, S. H. , and International Agency for Research on Cancer . 2008 WHO classification of tumours of haematopoietic and lymphoid tissues. International Agency for Research on Cancer, Lyon.

[2] Tamaru, J.‐I. 2017 2016 revision of the WHO classification of lymphoid neoplasms. Rinsho Ketsueki 58:2188–2193. 28978864 10.11406/rinketsu.58.2188

[3] Matutes, E. , K. Owusu‐Ankomah , R. Morilla , J. Garcia Marco , A. Houlihan , T. H. Que , et al. 1994 The immunological profile of B‐cell disorders and proposal of a scoring system for the diagnosis of CLL. Leukemia 8:1640–1645. 7523797

[4] Moreau, E. J. , E. Matutes , R. P. A'hern , A. M. Morilla , R. M. Morilla , K. A. Owusu‐Ankomah , et al. 1997 Improvement of the chronic lymphocytic leukemia scoring system with the monoclonal antibody SN8 (CD79b). Am. J. Clin. Pathol. 108:378–382. 9322589 10.1093/ajcp/108.4.378

[5] Binet, J. L. , A. Auquier , G. Dighiero , C. Chastang , H. Piguet , J. Goasguen , et al. 1981 A new prognostic classification of chronic lymphocytic leukemia derived from a multivariate survival analysis. Cancer 48:198–206. 7237385 10.1002/1097-0142(19810701)48:1<198::aid-cncr2820480131>3.0.co;2-v

[6] Rai, K. R. , A. Sawitsky , E. P. Cronkite , A. D. Chanana , R. N. Levy , and B. S. Pasternack . 1975 Clinical staging of chronic lymphocytic leukemia. Blood 46:219–234. 1139039

[7] Letestu, R. , V. Lévy , V. Eclache , F. Baran‐Marszak , D. Vaur , D. Naguib , et al. 2010 Prognosis of Binet stage A chronic lymphocytic leukemia patients: the strength of routine parameters. Blood 116:4588–4590. 20739656 10.1182/blood-2010-06-288274

[8] Damle, R. N. , T. Wasil , F. Fais , F. Ghiotto , A. Valetto , S. L. Allen , et al. 1999 Ig V gene mutation status and CD38 expression as novel prognostic indicators in chronic lymphocytic leukemia. Blood 94:1840–1847. 10477712

[9] Hamblin, T. J. , Z. Davis , A. Gardiner , D. G. Oscier , and F. K. Stevenson . 1999 Unmutated Ig V(H) genes are associated with a more aggressive form of chronic lymphocytic leukemia. Blood 94:1848–1854. 10477713

[10] Rossi, D. , A. Bruscaggin , V. Spina , S. Rasi , H. Khiabanian , M. Messina , et al. 2011 Mutations of the SF3B1 splicing factor in chronic lymphocytic leukemia: association with progression and fludarabine‐refractoriness. Blood 118:6904–6908. 22039264 10.1182/blood-2011-08-373159 PMC3245210

[11] Rigolin, G. M. , I. del Giudice , L. Formigaro , E. Saccenti , S. Martinelli , M. Cavallari , et al. 2015 Chromosome aberrations detected by conventional karyotyping using novel mitogens in chronic lymphocytic leukemia: clinical and biologic correlations. Genes Chromosom. Cancer 54:818–826. 26355802 10.1002/gcc.22293

[12] Rossi, D. , S. Rasi , V. Spina , A. Bruscaggin , S. Monti , C. Ciardullo , et al. 2013 Integrated mutational and cytogenetic analysis identifies new prognostic subgroups in chronic lymphocytic leukemia. Blood 121:1403–1412. 23243274 10.1182/blood-2012-09-458265 PMC3578955

[13] Gladstone, D. E. , L. Swinnen , Y. Kasamon , A. Blackford , C. D. Gocke , C. A. Griffin , et al. 2011 Importance of immunoglobulin heavy chain variable region mutational status in del(13q) chronic lymphocytic leukemia. Leuk. Lymphoma 52:1873–1881. 21851216 10.3109/10428194.2011.585529 PMC3790144

[14] Sandoval‐Sus, J. D. , J. C. Chavez , S. Dalia , S. M. H. Naqvi , C. Talati , L. Nodzon , et al. 2018 Association between immunoglobulin heavy‐chain variable region mutational status and isolated favorable baseline genomic aberrations in chronic lymphocytic leukemia. Leuk. Lymphoma 59:59–68. 28641468 10.1080/10428194.2017.1323271 PMC7771359

[15] Hallek, M. 2017 Chronic lymphocytic leukemia: 2017 update on diagnosis, risk stratification, and treatment. Am. J. Hematol. 92:946–965. 28782884 10.1002/ajh.24826

[16] Rosenquist, R. , D. Cortese , S. Bhoi , L. Mansouri , and R. Gunnarsson . 2013 Prognostic markers and their clinical applicability in chronic lymphocytic leukemia: where do we stand? Leuk. Lymphoma 54:2351–2364. 23480493 10.3109/10428194.2013.783913

[17] Gachard, N. , A. Salviat , C. Boutet , C. Arnoulet , F. Durrieu , B. Lenormand , et al. 2008 Multicenter study of ZAP‐70 expression in patients with B‐cell chronic lymphocytic leukemia using an optimized flow cytometry method. Haematologica 93:215–223. 18223290 10.3324/haematol.11622

[18] Forconi, F. , and P. Moss . 2015 Perturbation of the normal immune system in patients with CLL. Blood 126:573–581. 26084672 10.1182/blood-2015-03-567388

[19] Rizzo, D. , J. Chauzeix , F. Trimoreau , J. B. Woillard , F. Genevieve , A. Bouvier , et al. 2015 IgM peak independently predicts treatment‐free survival in chronic lymphocytic leukemia and correlates with accumulation of adverse oncogenetic events. Leukemia 29:337–345. 24943833 10.1038/leu.2014.198

[20] Parikh, S. A. , J. F. Leis , K. G. Chaffee , T. G. Call , C. A. Hanson , W. Ding , et al. 2015 Hypogammaglobulinemia in newly diagnosed chronic lymphocytic leukemia: natural history, clinical correlates, and outcomes. Cancer 121:2883–2891. 25931291 10.1002/cncr.29438 PMC4545721

[21] Martin, W. , R. Abraham , T. Shanafelt , R. J. Clark , N. Bone , S. M. Geyer , et al. 2007 Serum‐free light chain‐a new biomarker for patients with B‐cell non‐Hodgkin lymphoma and chronic lymphocytic leukemia. Transl. Res. 149:231–235. 17383597 10.1016/j.trsl.2006.11.001

[22] Xu, W. , Y. H. Wang , L. Fan , C. Fang , D. X. Zhu , D. M. Wang , et al. 2011 Prognostic significance of serum immunoglobulin paraprotein in patients with chronic lymphocytic leukemia. Leuk. Res. 35:1060–1065. 21208658 10.1016/j.leukres.2010.12.005

[23] Andersen, M. A. , F. J. Vojdeman , M. K. Andersen , P. D. N. Brown , C. H. Geisler , O. Weis Bjerrum , et al. 2016 Hypogammaglobulinemia in newly diagnosed chronic lymphocytic leukemia is a predictor of early death. Leuk. Lymphoma 57:1592–1599. 26850493 10.3109/10428194.2016.1142082

[24] Matutes, E. , and A. Polliack . 2016 A matter of life or early death in CLL: do serum immunoglobulin levels really count? Leuk. Lymphoma 57:1501–1502. 27118411 10.3109/10428194.2016.1170830

[25] Gachard, N. , M. Parrens , I. Soubeyran , B. Petit , A. Marfak , D. Rizzo , et al. 2013 IGHV gene features and MYD88 L265P mutation separate the three marginal zone lymphoma entities and Waldenström macroglobulinemia/lymphoplasmacytic lymphomas. Leukemia 27:183–189. 22944768 10.1038/leu.2012.257

[26] Bastard, C. , G. Raux , C. Fruchart , F. Parmentier , D. Vaur , D. Penther , et al. 2007 Comparison of a quantitative PCR method with FISH for the assessment of the four aneuploidies commonly evaluated in CLL patients. Leukemia 21:1460–1463. 17495973 10.1038/sj.leu.2404727

[27] Rizzo, D. , A. Lotay , N. Gachard , I. Marfak , J. L. Faucher , F. Trimoreau , et al. 2013 Very low levels of surface CD45 reflect CLL cell fragility, are inversely correlated with trisomy 12 and are associated with increased treatment‐free survival. Am. J. Hematol. 88:747–753. 23733486 10.1002/ajh.23494

[28] Del Giudice, I. , F. R. Mauro , M. S. De Propris , S. Santangelo , M. Marinelli , N. Peragine , et al. 2011 White blood cell count at diagnosis and immunoglobulin variable region gene mutations are independent predictors of treatment‐free survival in young patients with stage A chronic lymphocytic leukemia. Haematologica 96:626–630. 21193417 10.3324/haematol.2010.028779 PMC3069243

[29] Coordonné par Andrew Kramar et Simone Mathoulin‐Pélissier . Méthodes biostatistiques appliquées à la recherche clinique en cancérologie. Available at http://www.jle.com/fr/ouvrages/e-docs/methodes_biostatistiques_appliquees_a_la_recherche_clinique_en_cancerologie_290431/ouvrage.phtml (accessed 17 October 2017).

[30] Rosenquist, R. , A. Rosenwald , M. Q. Du , G. Gaidano , P. Groenen , A. Wotherspoon , et al. 2016 Clinical impact of recurrently mutated genes on lymphoma diagnostics: state‐of‐the‐art and beyond. Haematologica 101:1002–1009. 27582569 10.3324/haematol.2015.134510 PMC5060016

[31] International CLL‐IPI working group . 2016 An international prognostic index for patients with chronic lymphocytic leukaemia (CLL‐IPI): a meta‐analysis of individual patient data. Lancet Oncol. 17:779–790. 27185642 10.1016/S1470-2045(16)30029-8

[32] Gocke, C. D. , and D. E. Gladstone . 2018 The absolute percent deviation of IGHV mutation rather than a 98% cut‐off predicts survival of chronic lymphocytic leukaemia patients treated with fludarabine, cyclophosphamide and rituximab. Br. J. Haematol. 180:7–8. 29205267 10.1111/bjh.15015

[33] Delgado, J. , M. Doubek , T. Baumann , J. Kotaskova , S. Molica , P. Mozas , et al. 2017 Chronic lymphocytic leukemia: a prognostic model comprising only two biomarkers (IGHV mutational status and FISH cytogenetics) separates patients with different outcome and simplifies the CLL‐IPI. Am. J. Hematol. 92:375–380. 28120419 10.1002/ajh.24660

[34] Oppezzo, P. , C. Magnac , S. Bianchi , F. Vuillier , A. Tiscornia , G. Dumas , et al. 2002 Do CLL B cells correspond to naive or memory B‐lymphocytes? Evidence for an active Ig switch unrelated to phenotype expression and Ig mutational pattern in B‐CLL cells. Leukemia 16:2438–2446. 12454750 10.1038/sj.leu.2402731

