# Supplementary figures and images for: Normal serum protein electrophoresis and mutated IGHV genes detect very slowly evolving chronic lymphocytic leukemia patients
Source: Cancer Med. 2018 May 9;7(6):2621–8. doi: 10.1002/cam4.1510 (PMC6010869; doi:10.1002/cam4.1510)

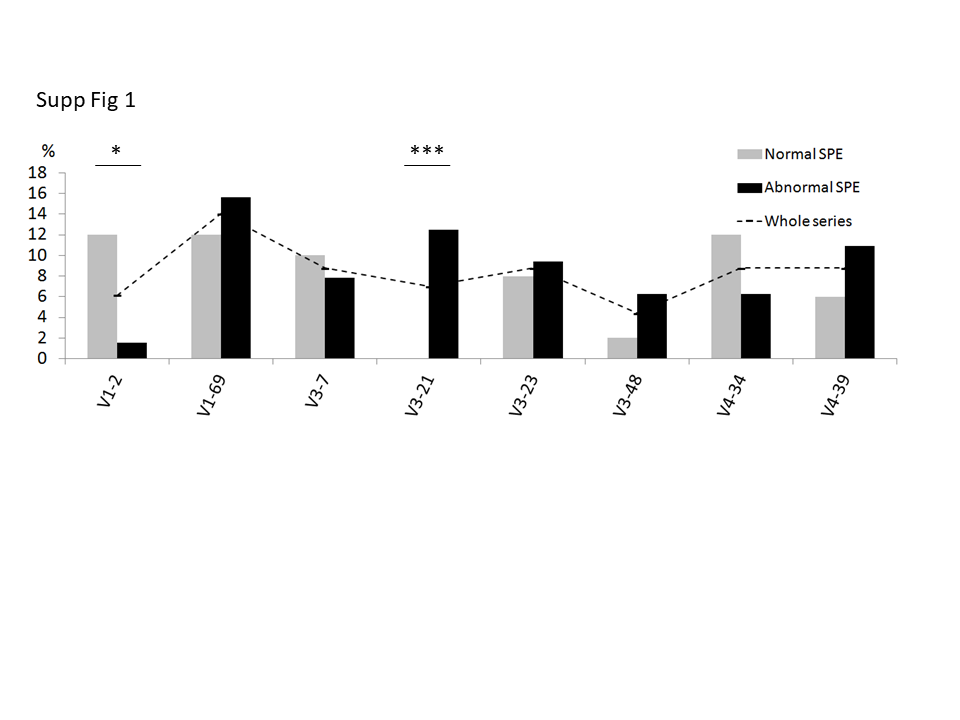

Supplement: Supplementary file 1 — Figure S1. IGHV repertoire‐Only IGHV gene rearrangements with a frequency over five percent of the whole series are shown (Chi2 test: P = 0.021 for IGHV1‐2 and P = 0.0095 for IGHV3‐21). [file CAM4-7-2621-s001.TIF]

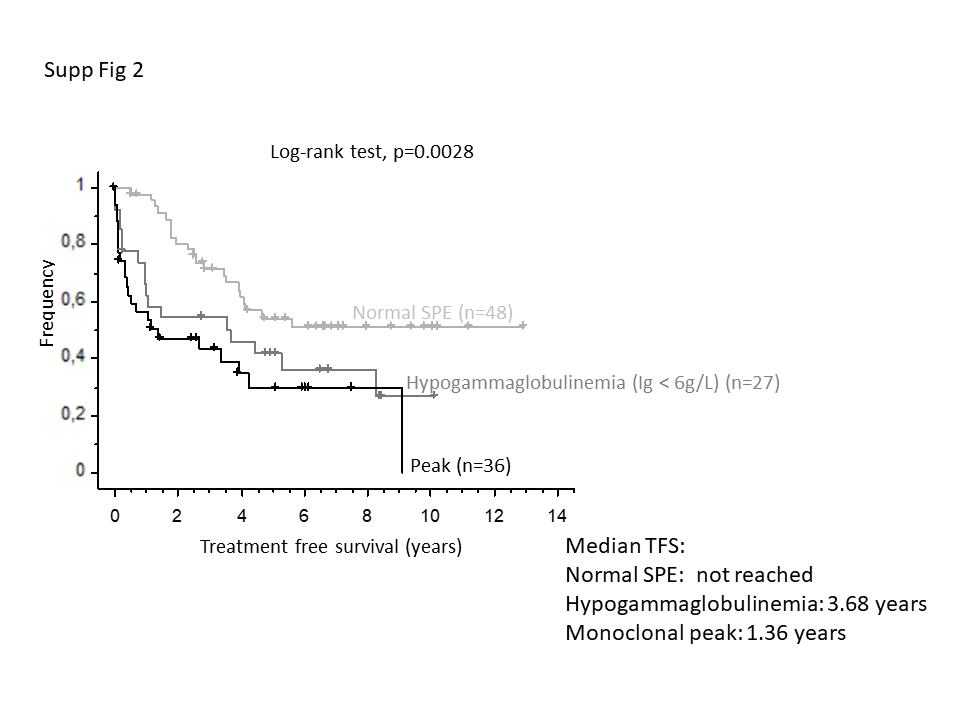

Supplement: Supplementary file 2 — Figure S2. Kaplan Meyer curves for treatment‐free survival for normal SPE, hypogammaglobulinemia and monoclonal peak. [file CAM4-7-2621-s002.TIF]

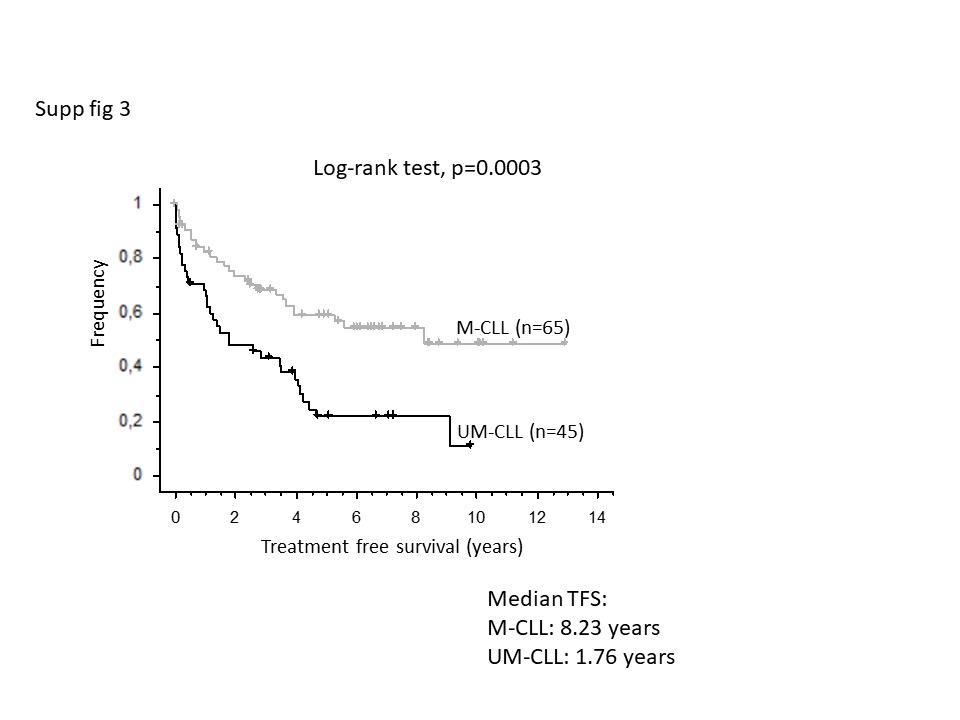

Supplement: Supplementary file 3 — Figure S3. Kaplan Meyer curves for treatment‐free survival according to IGHV mutation status. [file CAM4-7-2621-s003.TIF]

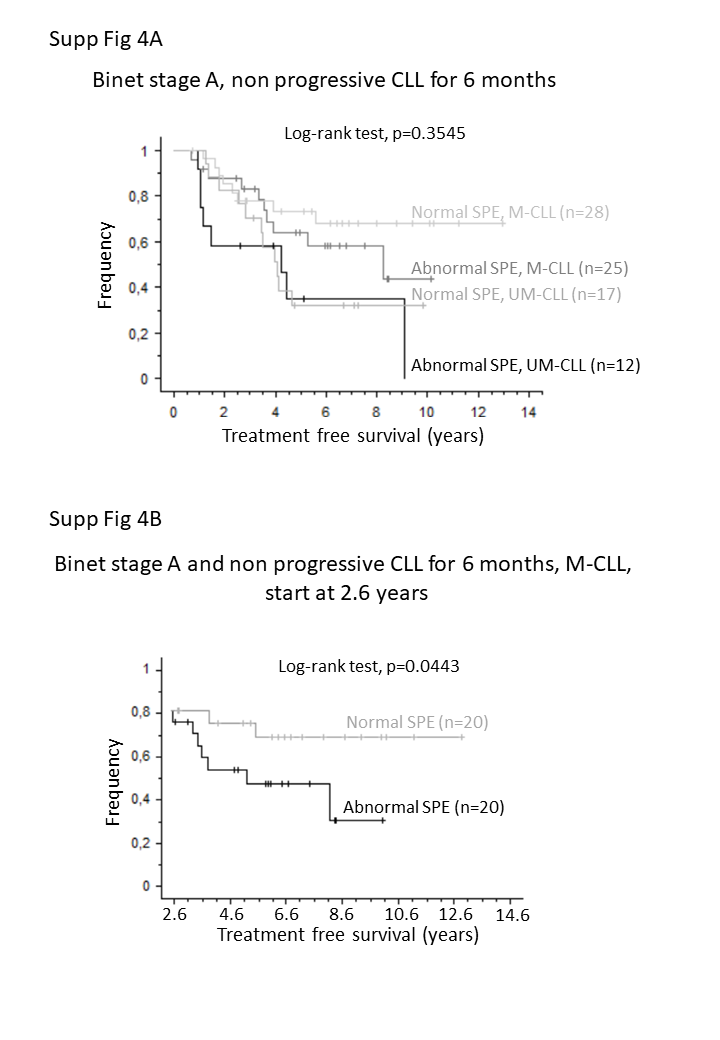

Supplement: Supplementary file 4 — Figure S4. Kaplan Meyer curves for treatment‐free survival (A) restricted to patients with non‐progressive CLL for 6 months and Binet stage A, according to SPE and IGHV mutation status. (B) Restricted to patients with non‐progressive CLL for 6 months, Binet stage A and mutated IGHV status, according to SPE, starting at 2.6 years). [file CAM4-7-2621-s004.TIF]

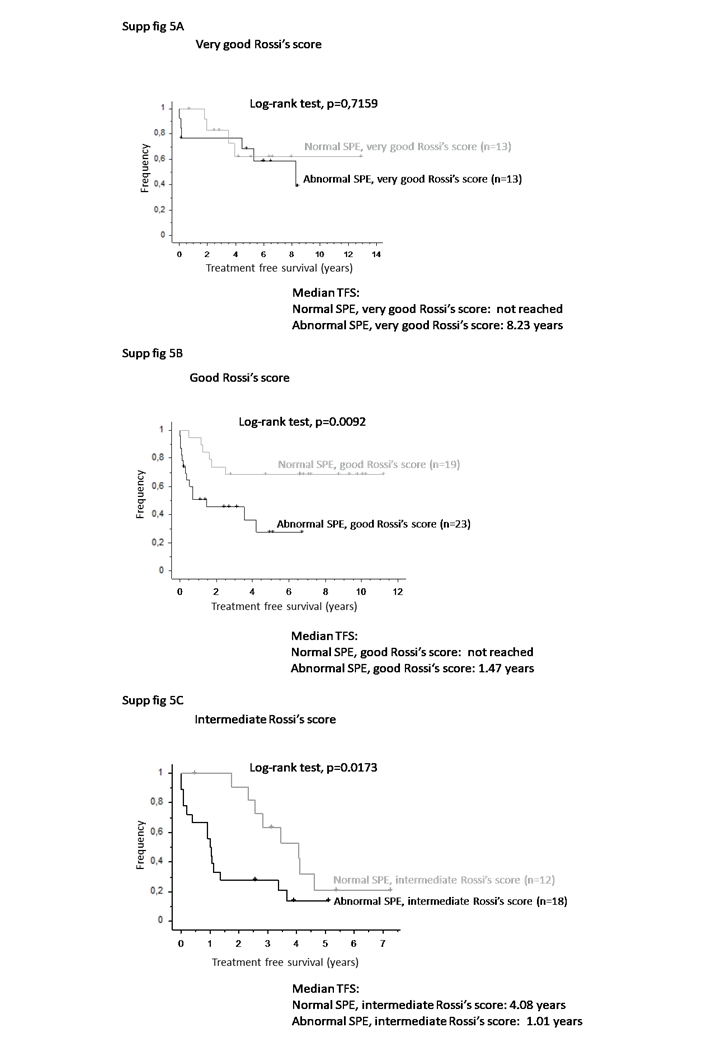

Supplement: Supplementary file 5 — Figure S5. Kaplan Meyer curves for treatment‐free survival according to SPE status and Rossi's score (A: very good Rossi's score. B: good Rossi's score. C: Intermediate Rossi's score). [file CAM4-7-2621-s005.TIF]

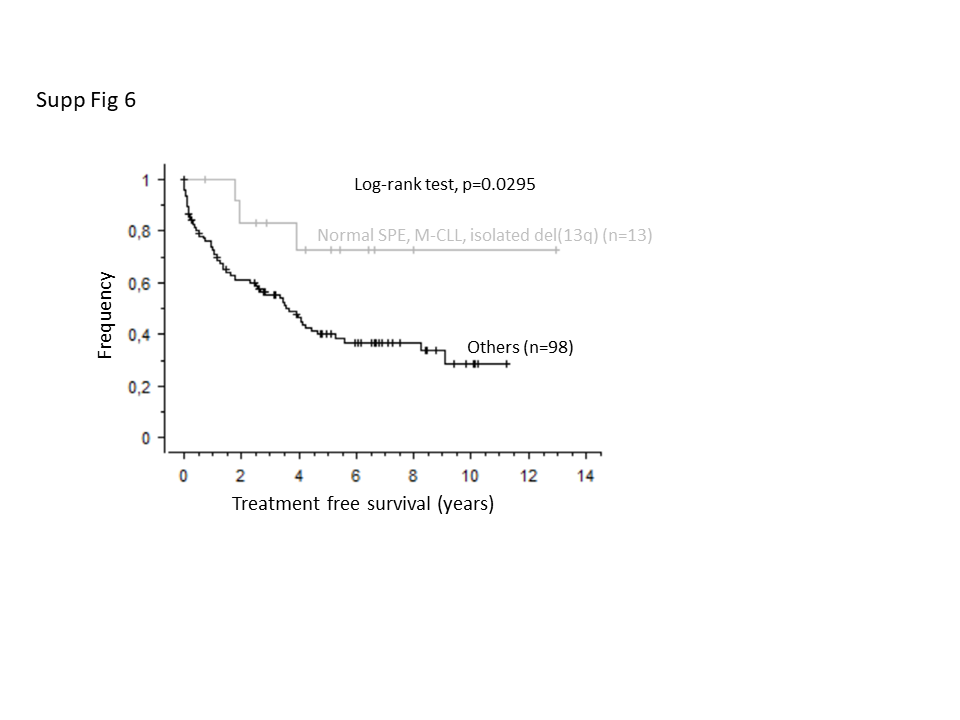

Supplement: Supplementary file 6 — Figure S6. Kaplan Meyer curves for treatment‐free survival for CLL patients with mutated IGHV genes, normal SPE and isolated del(13q) versus other patients of this whole series. [file CAM4-7-2621-s006.TIF]
